# Supplementary material for: Identification of a subset of immunosuppressive P2RX1-negative neutrophils in pancreatic cancer liver metastasis
Source: Nat Commun. 2021 Jan 8;12:174. doi: 10.1038/s41467-020-20447-y (PMC7794439; doi:10.1038/s41467-020-20447-y)
Supplement: Supplementary file 1 — Supplementary Information [file 41467_2020_20447_MOESM1_ESM.pdf]

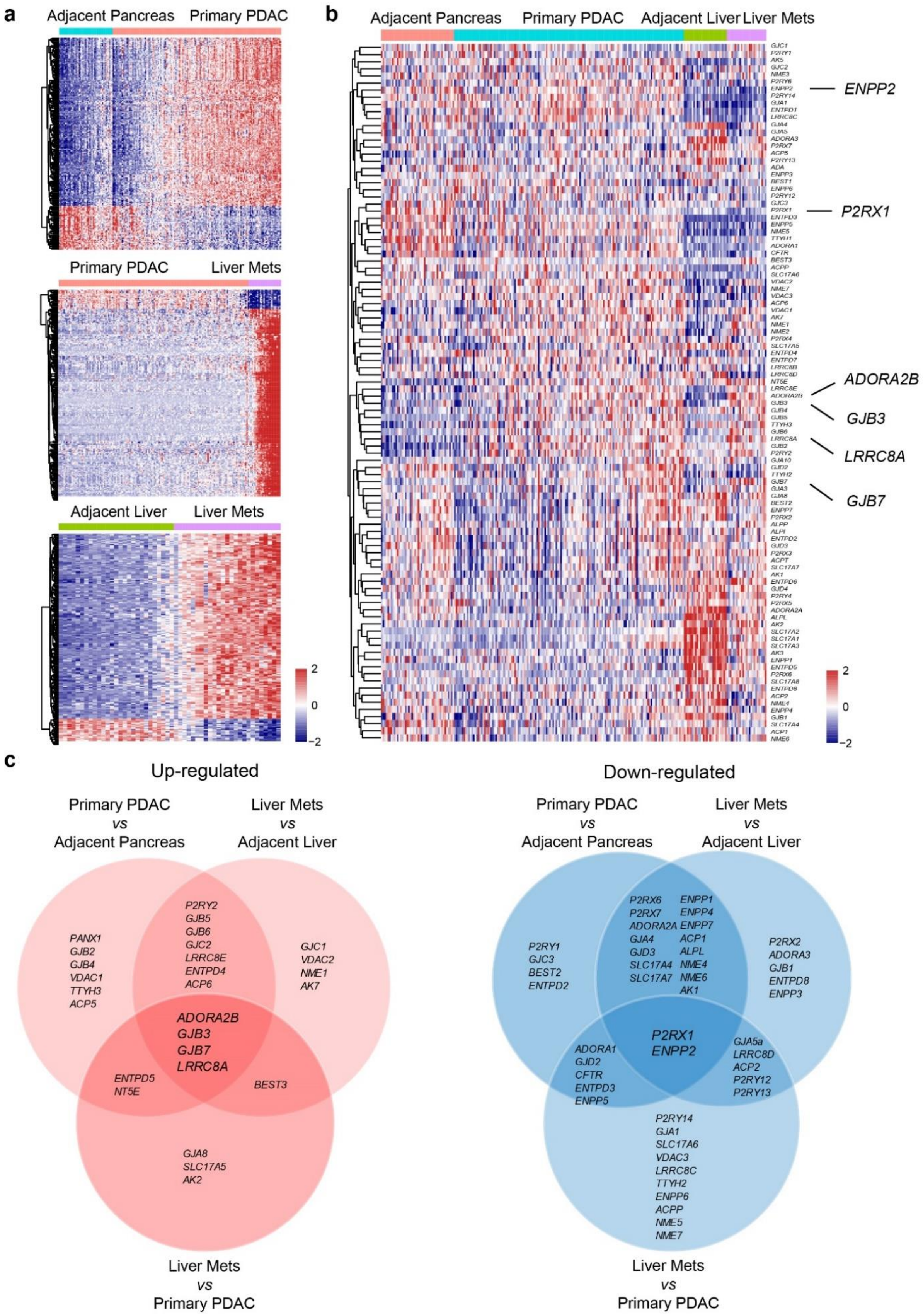

a
Adjacent Pancreas
Primary PDAC
Primary PDAC
Liver Mets
Adjacent Liver
Liver Mets
2
0
−2
b
Adjacent Pancreas
Primary PDAC
Adjacent Liver
Liver Mets
ENPP2
P2RX1
ADORA2B
GJB3
LRRC8A
GJB7
2
0
−2
c
Up-regulated
Primary PDAC vs Adjacent Pancreas
Liver Mets vs Adjacent Liver
PANX1 GJB2 GJB4 VDAC1 TTYH3 ACP5
P2RY2 GJB5 GJB6 GJC2 LRRC8E ENTPD4 ACP6
GJC1 VDAC2 NME1 AK7
ADORA2B GJB3 GJB7 LRRC8A
ENTPD5 NT5E
BEST3
GJA8 SLC17A5 AK2
Liver Mets vs Primary PDAC
Down-regulated
Primary PDAC vs Adjacent Pancreas
Liver Mets vs Adjacent Liver
P2RY1 GJC3 BEST2 ENTPD2
P2RX6 P2RX7 ADORA2A GJA4 GJD3 SLC17A4 SLC17A7
ENPP1 ENPP4 ENPP7 ACP1 ALPL NME4 NME6 AK1
P2RX2 ADORA3 GJB1 ENTPD8 ENPP3
P2RX1 ENPP2
ADORA1 GJD2 CFTR ENTPD3 ENPP5
GJA5a LRRC8D ACP2 P2RY12 P2RY13
P2RY14 GJA1 SLC17A6 VDAC3 LRRC8C TTYH2 ENPP6 ACPP NME5 NME7
Liver Mets vs Primary PDAC

Supplementary Fig. 1. Meta-analysis of clinical PDAC liver metastases. (a) Heatmap of differential gene expression between adjacent pancreas (n=46), primary PDAC (n=145), adjacent liver (n=27) and metastatic PDAC (n=25) samples. (b) Heatmap of purinergic signaling molecules expression in adjacent pancreas, primary PDAC, adjacent liver and metastatic PDAC samples. (c) Veen diagram of significantly up-regulated and down-regulated purinergic signaling molecules in adjacent pancreas, primary PDAC, adjacent liver and metastatic PDAC samples.

a

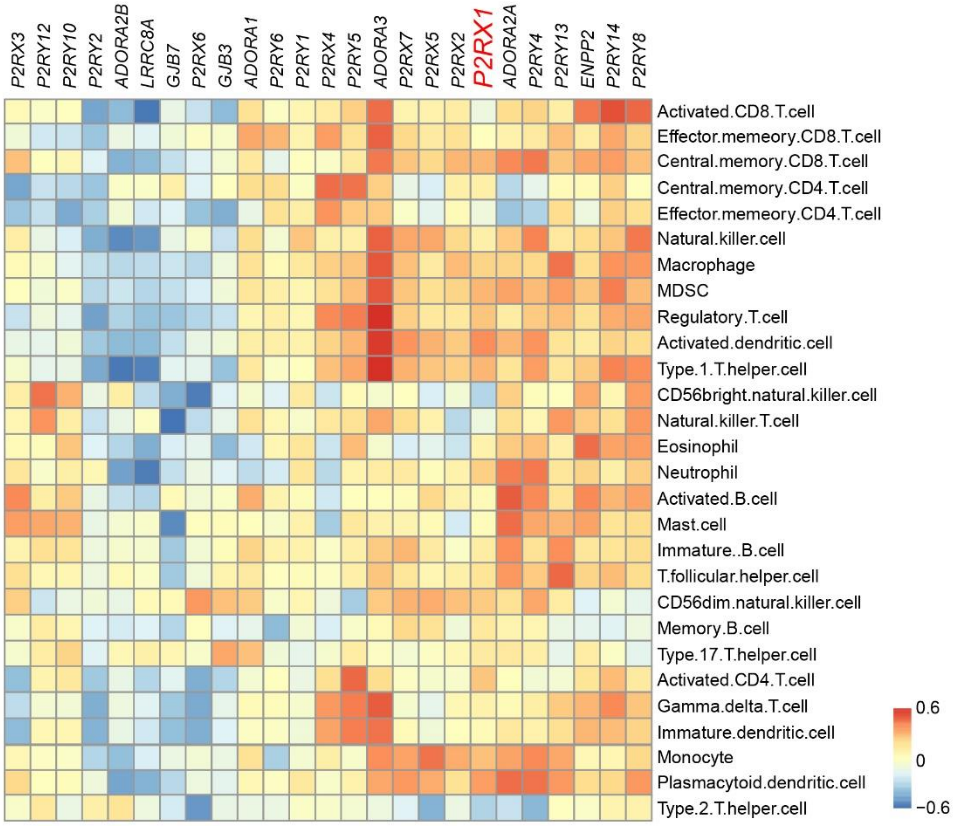

P2RX3
P2RY12
P2RY10
P2RY2
ADORA2B
LRRC8A
GJB7
P2RX6
GJB3
ADORA1
P2RY6
P2RY1
P2RX4
P2RY5
ADORA3
P2RX7
P2RX5
P2RX2
P2RX1
ADORA2A
P2RY4
P2RY13
ENPP2
P2RY14
P2RY8
Activated.CD8.T.cell
Effector.memeory.CD8.T.cell
Central.memory.CD8.T.cell
Central.memory.CD4.T.cell
Effector.memeory.CD4.T.cell
Natural.killer.cell
Macrophage
MDSC
Regulatory.T.cell
Activated.dendritic.cell
Type.1.T.helper.cell
CD56bright.natural.killer.cell
Natural.killer.T.cell
Eosinophil
Neutrophil
Activated.B.cell
Mast.cell
Immature..B.cell
T.follicular.helper.cell
CD56dim.natural.killer.cell
Memory.B.cell
Type.17.T.helper.cell
Activated.CD4.T.cell
Gamma.delta.T.cell
Immature.dendritic.cell
Monocyte
Plasmacytoid.dendritic.cell
Type.2.T.helper.cell
0.6
0
−0.6

b

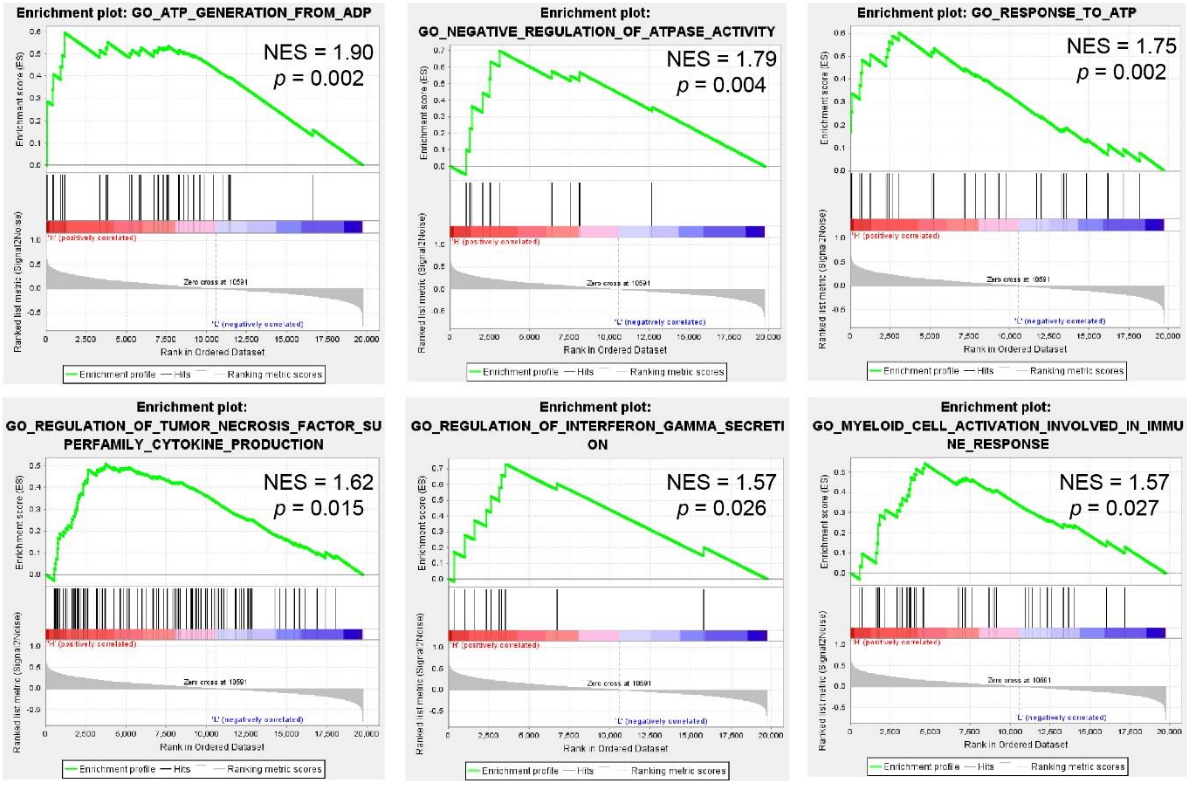

Enrichment plot: GO_ATP_GENERATION_FROM_ADP
NES = 1.90
p = 0.002
Enrichment plot: GO_NEGATIVE_REGULATION_OF_ATPASE_ACTIVITY
NES = 1.79
p = 0.004
Enrichment plot: GO_RESPONSE_TO_ATP
NES = 1.75
p = 0.002
Enrichment plot: GO_REGULATION_OF_TUMOR_NECROSIS_FACTOR_SUPERFAMILY_CYTOKINE_PRODUCTION
NES = 1.62
p = 0.015
Enrichment plot: GO_REGULATION_OF_INTERFERON_GAMMA_SECRETION
NES = 1.57
p = 0.026
Enrichment plot: GO_MYELOID_CELL_ACTIVATION_INVOLVED_IN_IMMUNE_RESPONSE
NES = 1.57
p = 0.027
Enrichment score (ES)
Ranked list metric (Signal2Noise)
Rank in Ordered Dataset
Enrichment profile
Hits
Ranking metric scores

Supplementary Fig. 2. Purinergic receptor P2RX1 was positively correlated with immune responses in liver metastatic PDAC. (a) Correlations between metastatic PDAC infiltrated 28 immune cell types and purinergic receptors + Veen diagram overlapped genes. (b) Gene set enrichment analysis comparing P2RX1-high with P2RX1-low expressing in metastatic PDAC samples. The *p* values and normalized enrichment score (NES) were shown. *P* values are derived from permutation test (b).

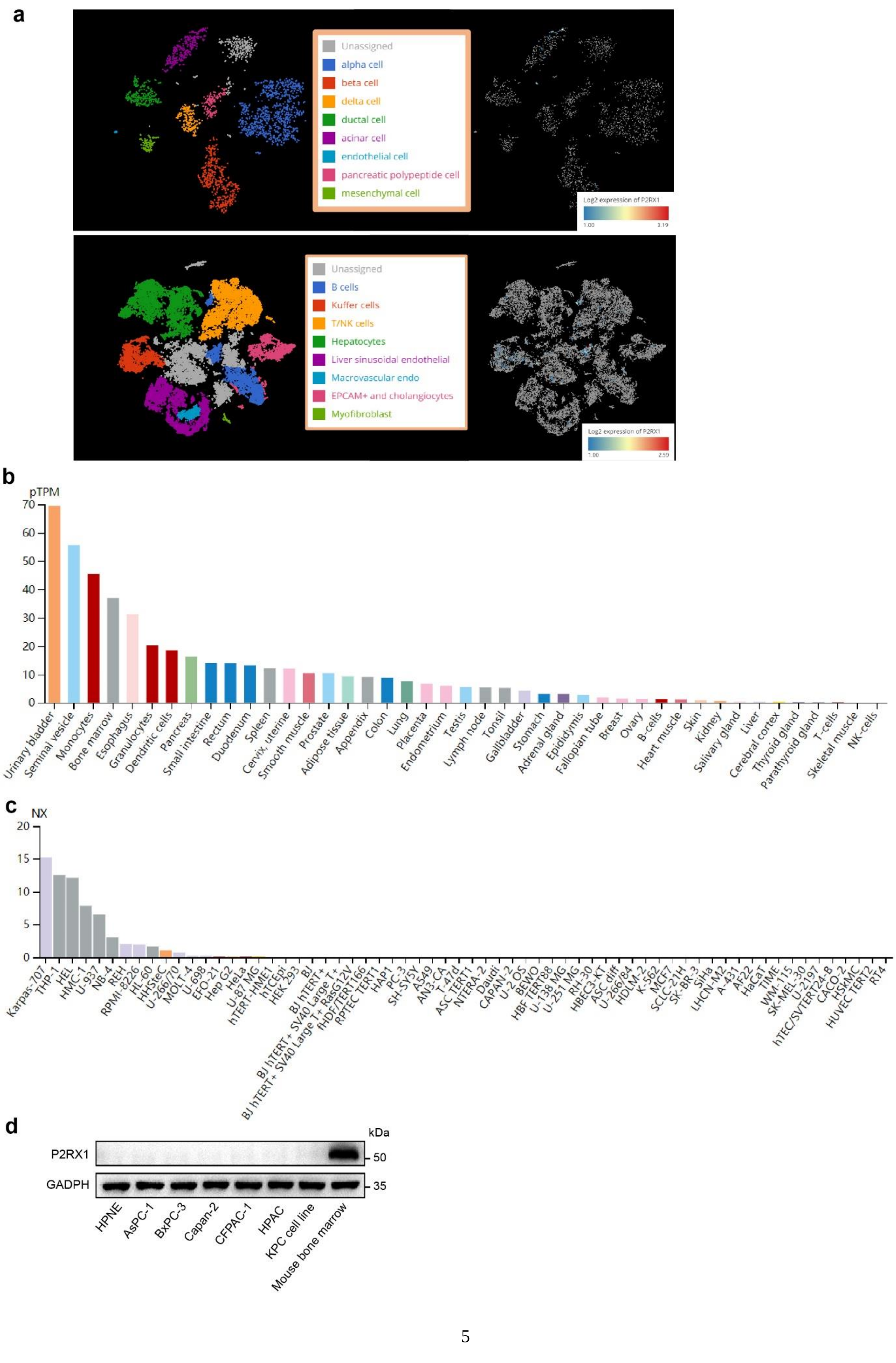

a
Unassigned
alpha cell
beta cell
delta cell
ductal cell
acinar cell
endothelial cell
pancreatic polypeptide cell
mesenchymal cell
Unassigned
B cells
Kuffer cells
T/NK cells
Hepatocytes
Liver sinusoidal endothelial
Macrovascular endo
EPCAM+ and cholangiocytes
Myofibroblast
b
pTPM
Urinary bladder
Seminal vesicle
Monocytes
Bone marrow
Esophagus
Granulocytes
Dendritic cells
Pancreas
Small intestine
Rectum
Duodenum
Spleen
Cervix, uterine
Smooth muscle
Prostate
Adipose tissue
Appendix
Colon
Lung
Placenta
Endometrium
Testis
Lymph node
Tonsil
Gallbladder
Stomach
Adrenal gland
Epididymis
Fallopian tube
Breast
Ovary
B-cells
Heart muscle
Skin
Kidney
Salivary gland
Liver
Cerebral cortex
Thyroid gland
Parathyroid gland
T-cells
Skeletal muscle
NK-cells
c
NX
Karpas-707
THP-1
HEL
HMC-1
U-937
NB-4
REH
RPMI-8226
HL-60
HHSteC
U-266/70
MOLT-4
U-698
EFO-21
Hep G2
HeLa
U-87 MG
hTERT-HME1
hTCEpi
HEK 293
BJ
BJ hTERT+
BJ hTERT+ SV40 Large T+
BJ hTERT+ SV40 Large T+ RasG12V
fHDF/TERT166
RPTEC TERT1
HAP1
PC-3
SH-SY5Y
A549
AN3-CA
T-47d
ASC TERT1
NTERA-2
Daudi
CAPAN-2
U-2 OS
BEWO
HBF TERT88
U-138 MG
U-251 MG
RH-30
HBEC3-KT
ASC diff
U-266/84
HDLM-2
K-562
MCF7
SCLC-21H
SK-BR-3
SiHa
LHCN-M2
A-431
AF22
HaCaT
TIME
WM-115
SK-MEL-30
U-2197
hTEC/SVTERT24-B
CACO-2
HSkMC
HUVEC TERT2
RT4
d
kDa
P2RX1
GADPH
50
35
HPNE
AsPC-1
BxPC-3
Capan-2
CFPAC-1
HPAC
KPC cell line
Mouse bone marrow

Supplementary Fig. 3. Systematic distribution of P2RX1. (a) Expression of P2RX1 in pancreas (upper) and liver (lower) tissues based on public signal cell sequencing data. (b) Expression of P2RX1 in different organs and leukocytes based on Human Protein Atlas (HPA) tissue RNA-seq database. (c) Expression of P2RX1 in different cell lines based on HPA cell line RNA-seq database. (d) Expression of P2RX1 in nonmalignant hTERT-HPNE pancreatic duct cells, PDAC cell lines and mouse bone marrow was determined by western blots (representative result from 4 independent experiments). Source data are provided as a Source Data file.

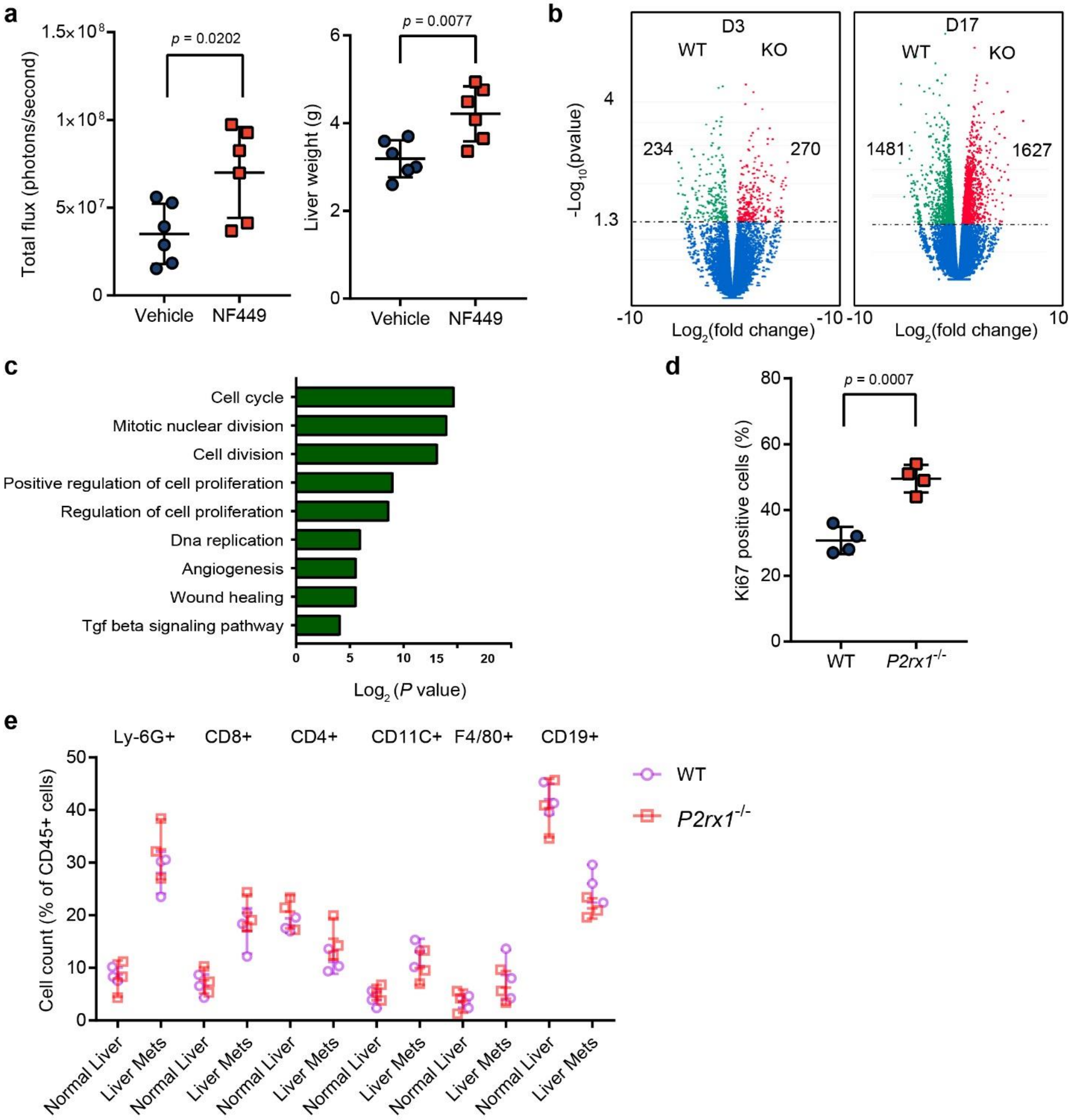

Supplementary Fig. 4. Influences of P2RX1 knockout on PDAC liver metastasis. (a) KPC cell was intrasplenically injected into WT mice and a selective P2RX1 antagonist, NF449, was administrated daily. At day 17, liver metastases were analyzed by *in vivo* imaging and liver weight. The graph showing the quantification results (n=6 per group, 2 independent experiments). (b-c) KPC cell was intrasplenically injected into WT or *P2rx1*$^{-/-}$ mice and two sequential stages (day 3

and 17) of liver metastases were harvested for RNA-seq (n=4 per group). Volcano plots of differential gene expression were shown in (b). GO Biological Process analysis of differential expressed genes between WT and *P2rx1*$^{-/-}$ at day 17 was performed in (c). (d) Quantitative analysis of Ki67 immunohistochemical staining (n=4 per group, 2 independent experiments). (e) Flow cytometry analyses of indicated immune cell types in WT and *P2rx1*$^{-/-}$ liver metastases at day17 (n=3 per group, 2 independent experiments). Bars represent mean ± standard deviation in a, d and e. *P* values are derived from two-sided Student's t test (a and d). Source data are provided as a Source Data file.

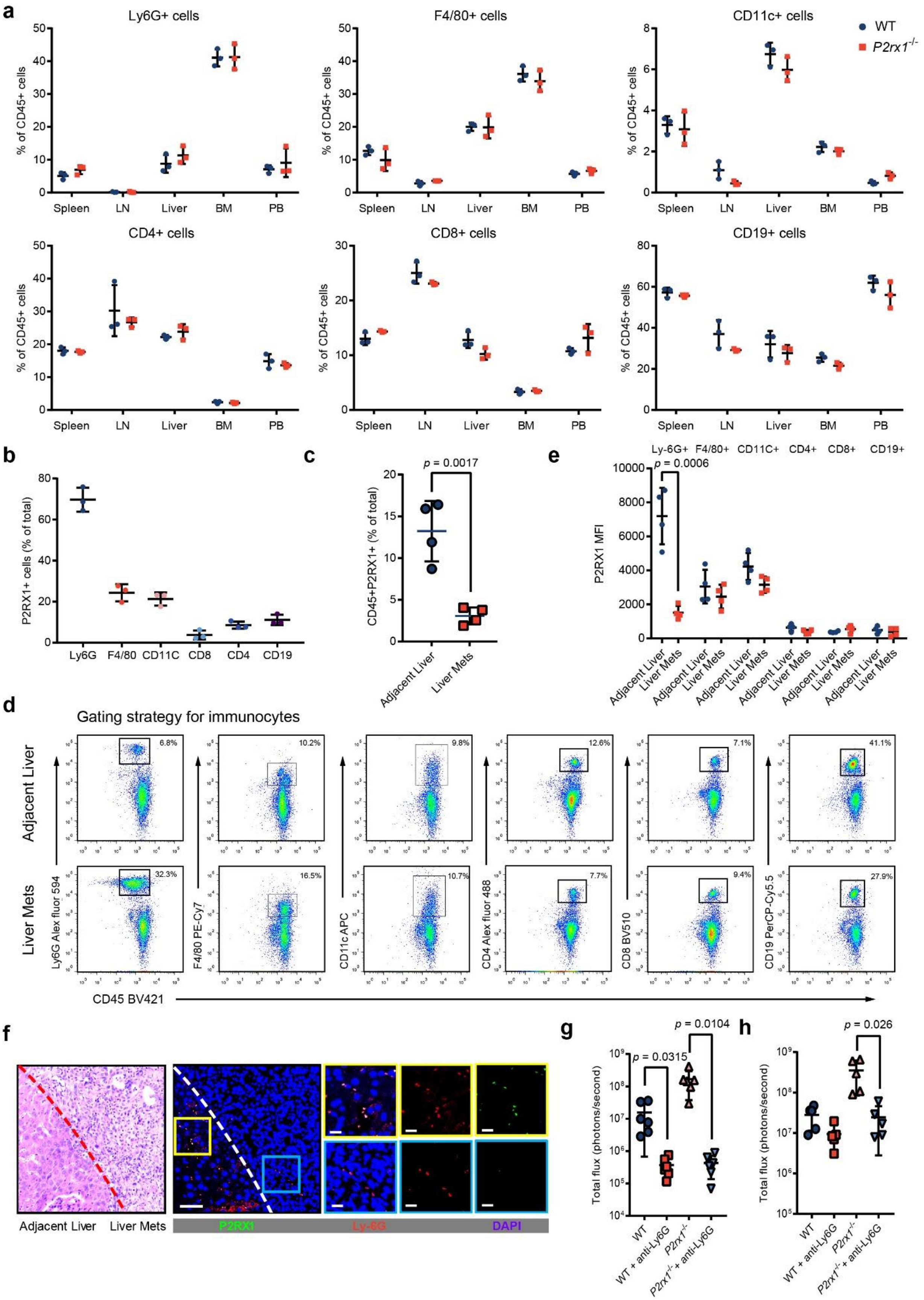

a
Ly6G+ cells
F4/80+ cells
CD11c+ cells
WT
P2rx1-/-
% of CD45+ cells
Spleen
LN
Liver
BM
PB
CD4+ cells
CD8+ cells
CD19+ cells
b
P2RX1+ cells (% of total)
Ly6G
F4/80
CD11C
CD8
CD4
CD19
c
p = 0.0017
CD45+P2RX1+ (% of total)
Adjacent Liver
Liver Mets
e
Ly-6G+
F4/80+
CD11C+
CD4+
CD8+
CD19+
p = 0.0006
P2RX1 MFI
d
Gating strategy for immunocytes
Adjacent Liver
Liver Mets
Ly6G Alex fluor 594
F4/80 PE-Cy7
CD11c APC
CD4 Alex fluor 488
CD8 BV510
CD19 PerCP-Cy5.5
CD45 BV421
f
Adjacent Liver
Liver Mets
P2RX1
Ly-6G
DAPI
g
p = 0.0315
p = 0.0104
Total flux (photons/second)
WT
WT + anti-Ly6G
P2rx1-/-
P2rx1-/- + anti-Ly6G
h
p = 0.026

Supplementary Fig. 5. Pro-tumoral P2RX1- neutrophils were accumulated in PDAC liver metastasis. (a) Immune cells in different organs of WT and *P2rx1*$^{-/-}$ mice were determined by flow cytometry (n=3 per group, 2 independent experiments). (b) Single cell suspension was obtained from mouse spleen and frequency of P2RX1+ cells was determined in indicated immune cell types (n=3 per group, 2 independent experiments). (c) KPC cells were intrasplenically injected to seed livers of WT mice. Single cell suspension was obtained from liver metastases and adjacent livers of WT mice at day 17. Frequency of CD45+P2RX1+ cells was determined by flow cytometry (n=4 per group, 3 independent experiments). (d-e) KPC cells were intrasplenically injected to seed livers of WT mice. Immune cells were enriched from single cell suspension of liver metastases and adjacent liver tissues at day 17. P2RX1 expression in the indicated immune cell types was determined by flow cytometry. Gating strategy of indicated immune cells was shown in d, and mean fluorescence intensity (MFI) of P2RX1 was shown in e (n=4 per group, 3 independent experiments). (f) Representative images of H&E staining and immunofluorescence staining of P2RX1 (Green), Ly6G (Red) and DAPI (Blue) in KPC cell intrasplenical injection induced liver metastases (representative result from 6 experiments). 50 μm of scale bar for low power fields, 20 μm of scale bar for high power fields. (g-h) KPC cells were intrasplenically injected to seed livers of WT and *P2rx1*$^{-/-}$ mice. Neutrophils in WT and *P2rx1*$^{-/-}$ mice were depleted by intraperitoneal injection of anti-Ly6G (clone 1A8) antibody 1 day before or 4 days after injection of KPC cells. At day 17, liver metastases were analyzed by *in vivo* imaging. The graph showing the quantification results (n=5-6 per group, 2 independent experiments). Bars represent mean ± standard deviation in a-c, e, g and h. *P* values are derived from two-sided Student's t test (c and e), or one-way ANOVA and Tukey's multiple comparisons test (g and h). Source data are provided as a Source Data file.

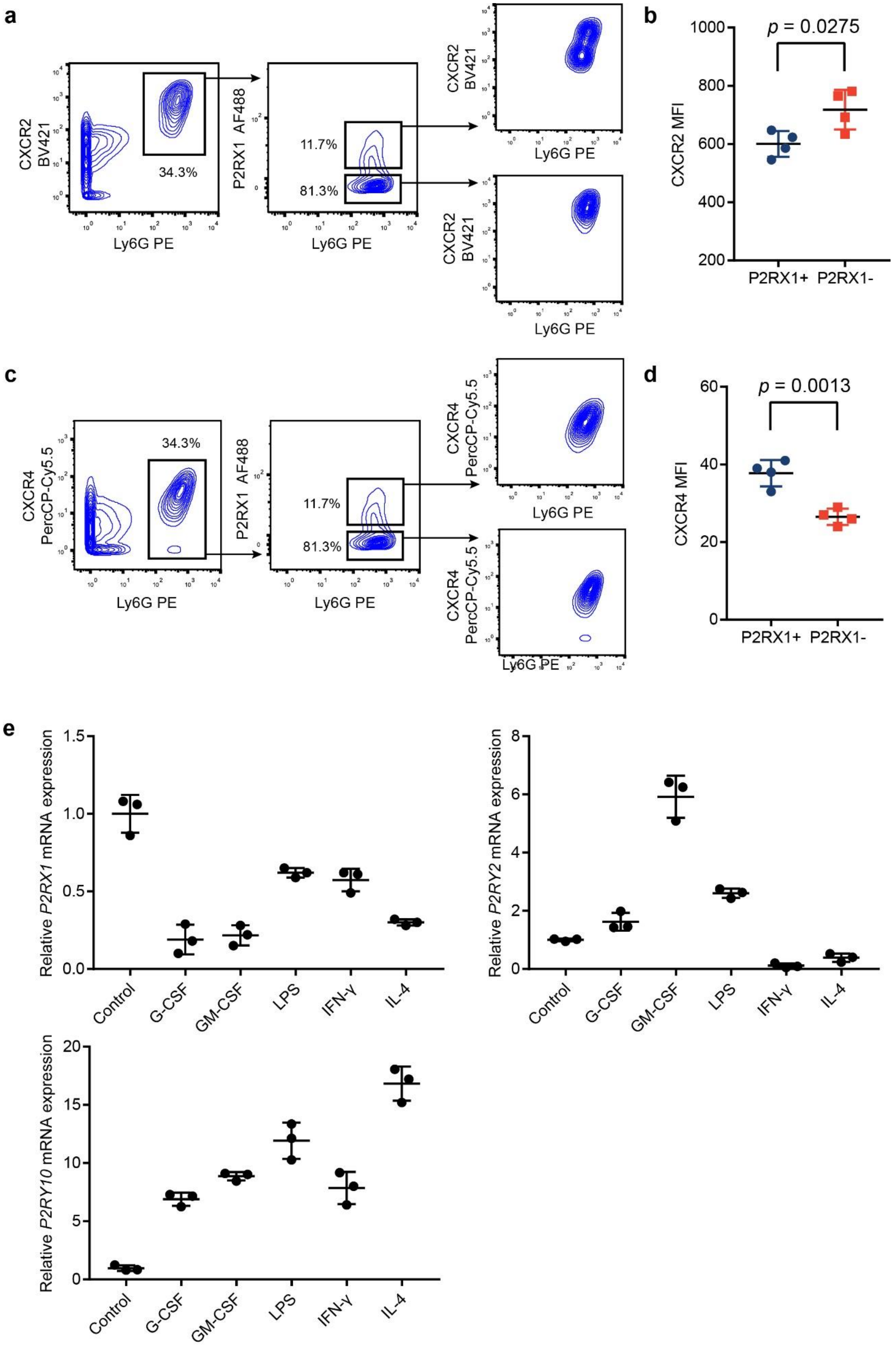
a
CXCR2 BV421
34.3%
Ly6G PE
P2RX1 AF488
11.7%
81.3%
Ly6G PE
CXCR2 BV421
Ly6G PE
CXCR2 BV421
Ly6G PE
b
p = 0.0275
CXCR2 MFI
1000
800
600
400
200
P2RX1+
P2RX1-
c
CXCR4 PercCP-Cy5.5
34.3%
Ly6G PE
P2RX1 AF488
11.7%
81.3%
Ly6G PE
CXCR4 PercCP-Cy5.5
Ly6G PE
CXCR4 PercCP-Cy5.5
Ly6G PE
d
p = 0.0013
CXCR4 MFI
60
40
20
0
P2RX1+
P2RX1-
e
Relative P2RX1 mRNA expression
1.5
1.0
0.5
0.0
Control
G-CSF
GM-CSF
LPS
IFN-γ
IL-4
Relative P2RY2 mRNA expression
8
6
4
2
0
Control
G-CSF
GM-CSF
LPS
IFN-γ
IL-4
Relative P2RY10 mRNA expression
20
15
10
5
0
Control
G-CSF
GM-CSF
LPS
IFN-γ
IL-4

Supplementary Fig. 6. PDAC liver metastasis systematically mobilizes and recruits P2RX1-neutrophils. (a-d) KPC cell was intrasplenically injected to seed livers of WT mice. Single cell suspension was obtained from liver metastases at day 17. CXCR2 (a-b) and CXCR4 (c-d) expression on neutrophils were determined by flow cytometry and quantitative results were shown (n=4 per group, 2 independent experiments). (e) Bone marrow neutrophils were isolated from WT mice and stimulated with indicated stimulus. RT-qPCR was performed to detect the mRNA expression of *P2rx1*, *P2ry2*, and *P2ry10* (n=3 per group, 2 independent experiments). Bars represent mean ± standard deviation in b, d and e. *P* values are derived from two-sided Student's t test (b and d). Source data are provided as a Source Data file.

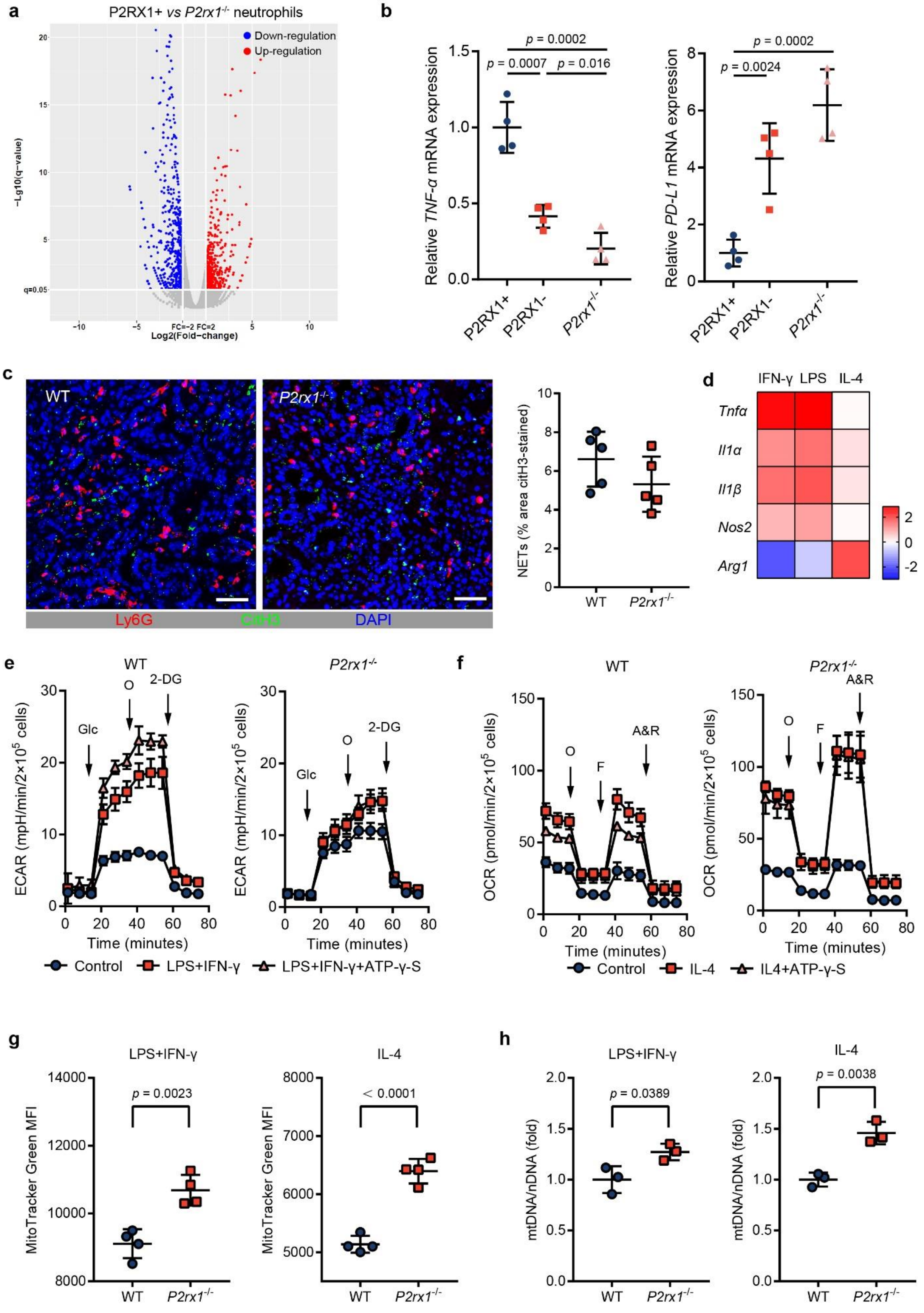

a
P2RX1+ vs P2rx1-/- neutrophils
Down-regulation
Up-regulation
−Lg10(q−value)
Log2(Fold−change)
q=0.05
FC=-2 FC=2
b
Relative TNF-α mRNA expression
p = 0.0002
p = 0.0007
p = 0.016
Relative PD-L1 mRNA expression
p = 0.0024
P2RX1+
P2RX1-
P2rx1-/-
c
WT
P2rx1-/-
Ly6G
CitH3
DAPI
NETs (% area citH3-stained)
d
IFN-γ
LPS
IL-4
Tnfα
Il1α
Il1β
Nos2
Arg1
e
Glc
O
2-DG
ECAR (mpH/min/2×10^5 cells)
Time (minutes)
Control
LPS+IFN-γ
LPS+IFN-γ+ATP-γ-S
f
F
A&R
OCR (pmol/min/2×10^5 cells)
IL-4
IL4+ATP-γ-S
g
p = 0.0023
< 0.0001
MitoTracker Green MFI
h
p = 0.0389
p = 0.0038
mtDNA/nDNA (fold)

Supplementary Fig. 7. Characterizing the heterogeneous features of *P2rx1*$^{-/-}$ and P2RX1+ neutrophils. (a) KPC cells were intrasplenically injected to seed livers of WT and *P2rx1*$^{-/-}$ mice. Single cell suspension was obtained from liver metastases at day 17. Then, P2RX1+ neutrophils were purified from WT mice, and *P2rx1*$^{-/-}$ neutrophils were purified from *P2rx1*$^{-/-}$ mice for RNA sequencing. Volcano plots of differential gene expression were shown (n=1 per group). (b) KPC cells were intrasplenically injected to seed livers of WT and *P2rx1*$^{-/-}$ mice. Single cell suspension was obtained from liver metastases at day 17. P2RX1+ and P2RX1- neutrophils were purified from WT mice, and *P2rx1*$^{-/-}$ neutrophils were purified from *P2rx1*$^{-/-}$ mice. RT-qPCR was performed to detected the mRNA expression of *TNF-α* and *PD-L1* (n=4 per group, 2 independent experiments). (c) KPC cells were intrasplenically injected to seed livers of WT and *P2rx1*$^{-/-}$ mice. At day 17, liver metastatic tissues were harvested from WT and *P2rx1*$^{-/-}$ mice. Ly6G (Red), citrullinated histone H3 (CitH3) (Green) and DAPI (Blue) were stained to determine neutrophil extracellular traps (NETs) (n=4 per group, 2 independent experiments). The scale bar is 50 μm. (d) BM was isolated from WT mice and stimulated with indicated stimulus. RNA-seq were performed and Log_2 fold change compared to control were shown (n=1 per group). (e-f) Bone marrow neutrophils were isolated from WT and *P2rx1*$^{-/-}$ mice and stimulated with LPS+IFN-γ in the presence or absence of ATP-γ-S. The ECAR was then measured by a Seahorse assay (n=4 per group, 2 independent experiments). Glc, glucose; O, oligomycin; 2-DG, 2-deoxyglucose. (f) Bone marrow neutrophils were isolated from WT and *P2rx1*$^{-/-}$ mice and stimulated with IL-4 in the presence or absence of ATP-γ-S. The OCR was then measured by a Seahorse assay (n=4 per group, 2 independent experiments). O, oligomycin; F, FCCP (carbonyl cyanide 4-[trifluoromethoxy] phenylhydrazone); A & R, antimycin A and rotenone. (g) BM was isolated from WT or *P2rx1*$^{-/-}$ mice and stimulated with indicated stimulus. Mitotracker Green was used to determine mitochondrial mass with flow

cytometry (n=3 per group, 3 independent experiments). (h) BM was isolated from WT or *P2rx1*$^{-/-}$ mice and stimulated with indicated stimulus. Mitochondrial DNA (mtDNA)/nuclear DNA (nDNA) ratio was determined by PCR (n=3 per group, 3 independent experiments). Bars represent mean ± standard deviation in b, c and e-h. *P* values are derived from two-sided Student's t test (b, g and h). Source data are provided as a Source Data file.

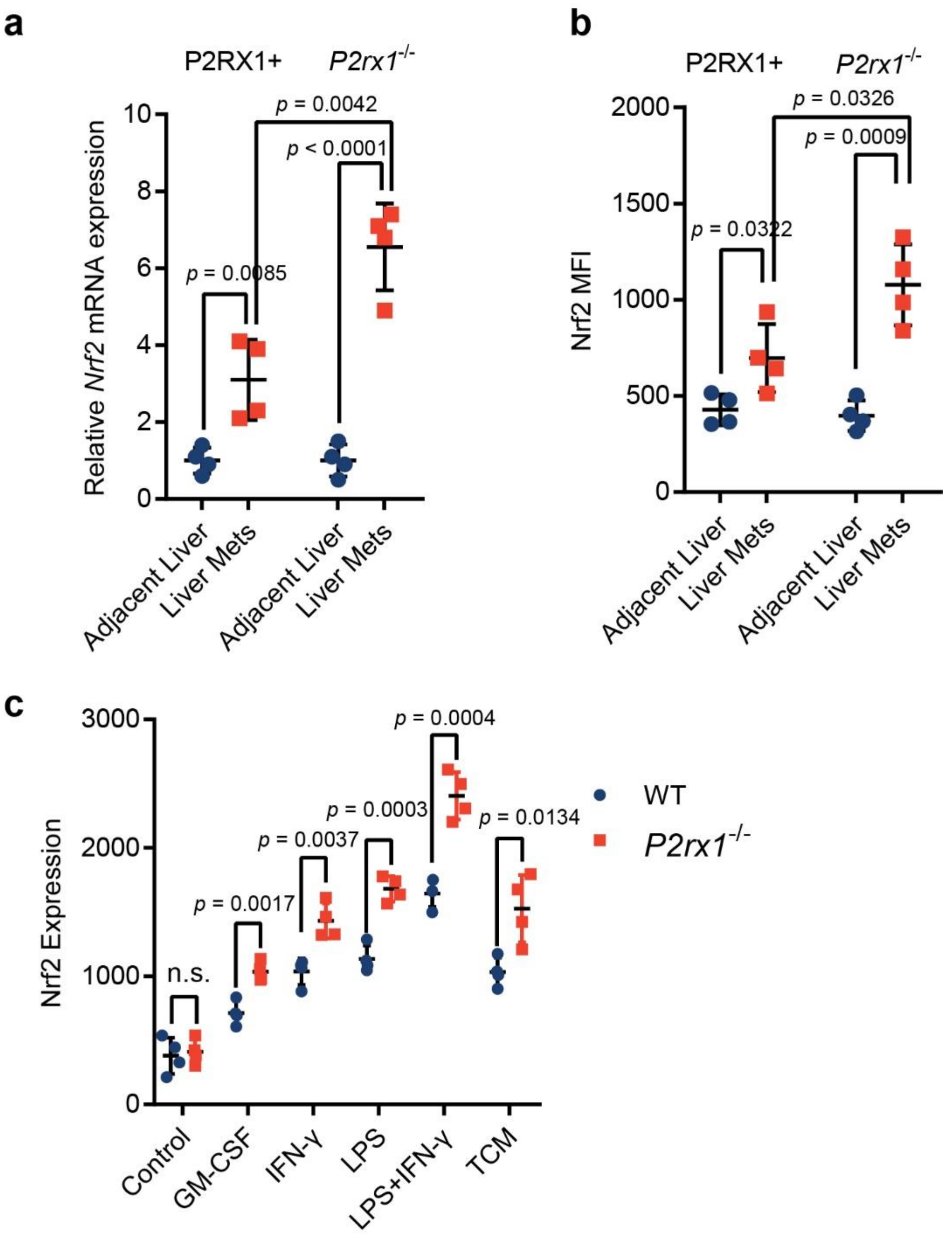

Supplementary Fig. 8. Up-regulated Nrf2 is essential for shaping immune-tolerant phenotype of P2RX1 KO neutrophils. (a and b) KPC cells were intrasplenically injected to seed livers of WT and *P2rx1*$^{-/-}$ mice. Single cell suspension was obtained from liver metastases at day 17. Then, P2RX1+ neutrophils were purified from WT mice, and *P2rx1*$^{-/-}$ neutrophils were purified from *P2rx1*$^{-/-}$ mice. *Nrf2* mRNA was determined by qPCR (a) and Nrf2 protein was determined by flow cytometry (b) (n=4 per group, 3 independent experiments). (c) Bone marrow neutrophils

were isolated from WT or *P2rx1*$^{-/-}$ mice and stimulated with indicated stimulus and an inhibitor Nrf2 inhibitor. Nrf2 expression was measured by flow cytometry (n=4 per group, 3 independent experiments). Bars represent mean ± standard deviation in a-c. *P* values are derived from one-way ANOVA and Tukey's multiple comparisons test (a and b), or two-sided Student's t test (c). Source data are provided as a Source Data file.

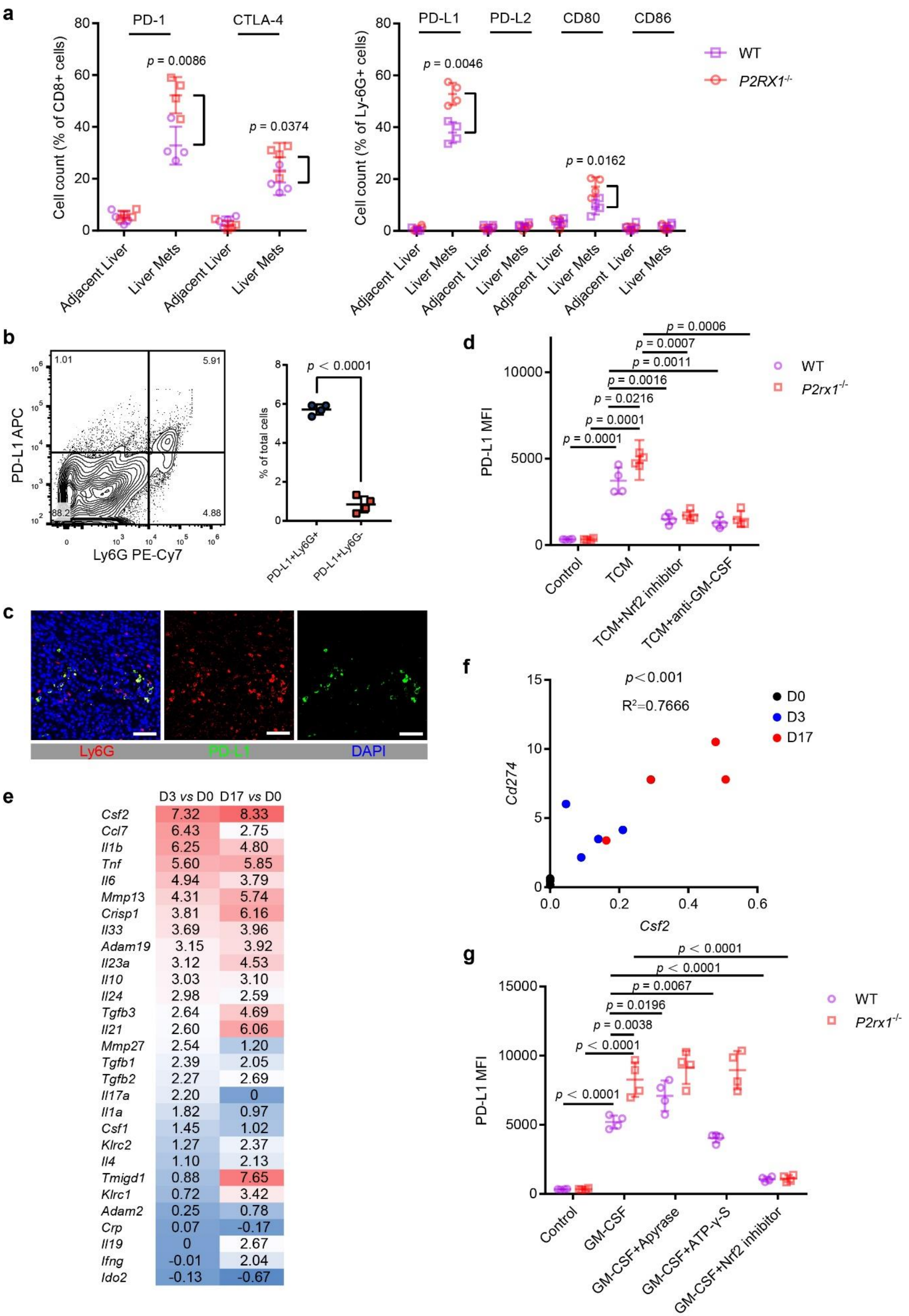
a
PD-1
CTLA-4
p = 0.0086
p = 0.0374
Cell count (% of CD8+ cells)
Adjacent Liver
Liver Mets
Adjacent Liver
Liver Mets
PD-L1
PD-L2
CD80
CD86
p = 0.0046
p = 0.0162
Cell count (% of Ly-6G+ cells)
WT
P2RX1-/-
b
1.01
5.91
88.2
4.88
PD-L1 APC
Ly6G PE-Cy7
p < 0.0001
% of total cells
PD-L1+Ly6G+
PD-L1+Ly6G-
c
Ly6G
PD-L1
DAPI
d
p = 0.0006
p = 0.0007
p = 0.0011
p = 0.0016
p = 0.0216
p = 0.0001
p = 0.0001
PD-L1 MFI
WT
P2rx1-/-
Control
TCM
TCM+Nrf2 inhibitor
TCM+anti-GM-CSF
e
D3 vs D0 | D17 vs D0
Csf2 7.32 8.33
Ccl7 6.43 2.75
Il1b 6.25 4.80
Tnf 5.60 5.85
Il6 4.94 3.79
Mmp13 4.31 5.74
Crisp1 3.81 6.16
Il33 3.69 3.96
Adam19 3.15 3.92
Il23a 3.12 4.53
Il10 3.03 3.10
Il24 2.98 2.59
Tgfb3 2.64 4.69
Il21 2.60 6.06
Mmp27 2.54 1.20
Tgfb1 2.39 2.05
Tgfb2 2.27 2.69
Il17a 2.20 0
Il1a 1.82 0.97
Csf1 1.45 1.02
Klrc2 1.27 2.37
Il4 1.10 2.13
Tmigd1 0.88 7.65
Klrc1 0.72 3.42
Adam2 0.25 0.78
Crp 0.07 -0.17
Il19 0 2.67
Ifng -0.01 2.04
Ido2 -0.13 -0.67
f
p<0.001
R2=0.7666
D0
D3
D17
Cd274
Csf2
g
p < 0.0001
p < 0.0001
p = 0.0067
p = 0.0196
p = 0.0038
p < 0.0001
p < 0.0001
PD-L1 MFI
WT
P2rx1-/-
Control
GM-CSF
GM-CSF+Apyrase
GM-CSF+ATP-γ-S
GM-CSF+Nrf2 inhibitor

Supplementary Fig. 9. GM-CSF promotes neutrophil PD-L1 expression in PDAC liver metastasis. (a) Single cell suspension was obtained from liver metastases of WT and *P2rx1*$^{-/-}$ mice at day 17. Flow cytometry was performed to detect the frequency of indicated markers (n=4 per group, 3 independent experiments). (b) Single cell suspension was obtained from liver metastases of *P2rx1*$^{-/-}$ mice at day 17. PD-L1 and Ly6G expression was detected by flow cytometry (n=4 per group, 3 independent experiments). (c) Representative images of immunofluorescence staining of Ly6G (Red), PD-L1 (Green) and DAPI (Blue) in KPC cell intrasplenical injection induced liver metastases (representative result from 4 independent experiments). The scale bar is 50 μm. (d) Bone marrow neutrophils isolated from WT and *P2rx1*$^{-/-}$ mice were stimulated with tumor conditioned medium (TCM) in the presence of a Nrf2 inhibitor or anti-GM-CSF neutralizing antibody. PD-L1 expression was detected by flow cytometry (n=4 per group, 3 independent experiments). (e) Inflammation-associated cytokines and chemokines in murine PDAC liver metastases were screened based on RNA-seq data obtained from liver metastases of WT mice. Log_2 fold change of D3 and D17 as compared to day 0 was shown (n=3 for D0, and n=4 for D3 and D17, 1 independent experiments). (f) Correlation between Cd274 (PD-L1) and Csf2 (GM-CSF) was analyzed based on RNA-seq data obtained from liver metastases of WT and *P2rx1*$^{-/-}$ mice (n=3 for D0, and n=4 for D3 and D17, 1 independent experiments). (g) Bone marrow neutrophils were isolated from WT or *P2rx1*$^{-/-}$ mice and stimulated with indicated stimulus. PD-L1 expression was detected by flow cytometry (n=4 per group, 3 independent experiments). Bars represent mean ± standard deviation in a, b, d, f and g. *P* values are derived from two-sided Student's t test (a-b), one-way ANOVA and Tukey's multiple comparisons test (d and g), or Spearman's test (f). Source data are provided as a Source Data file.

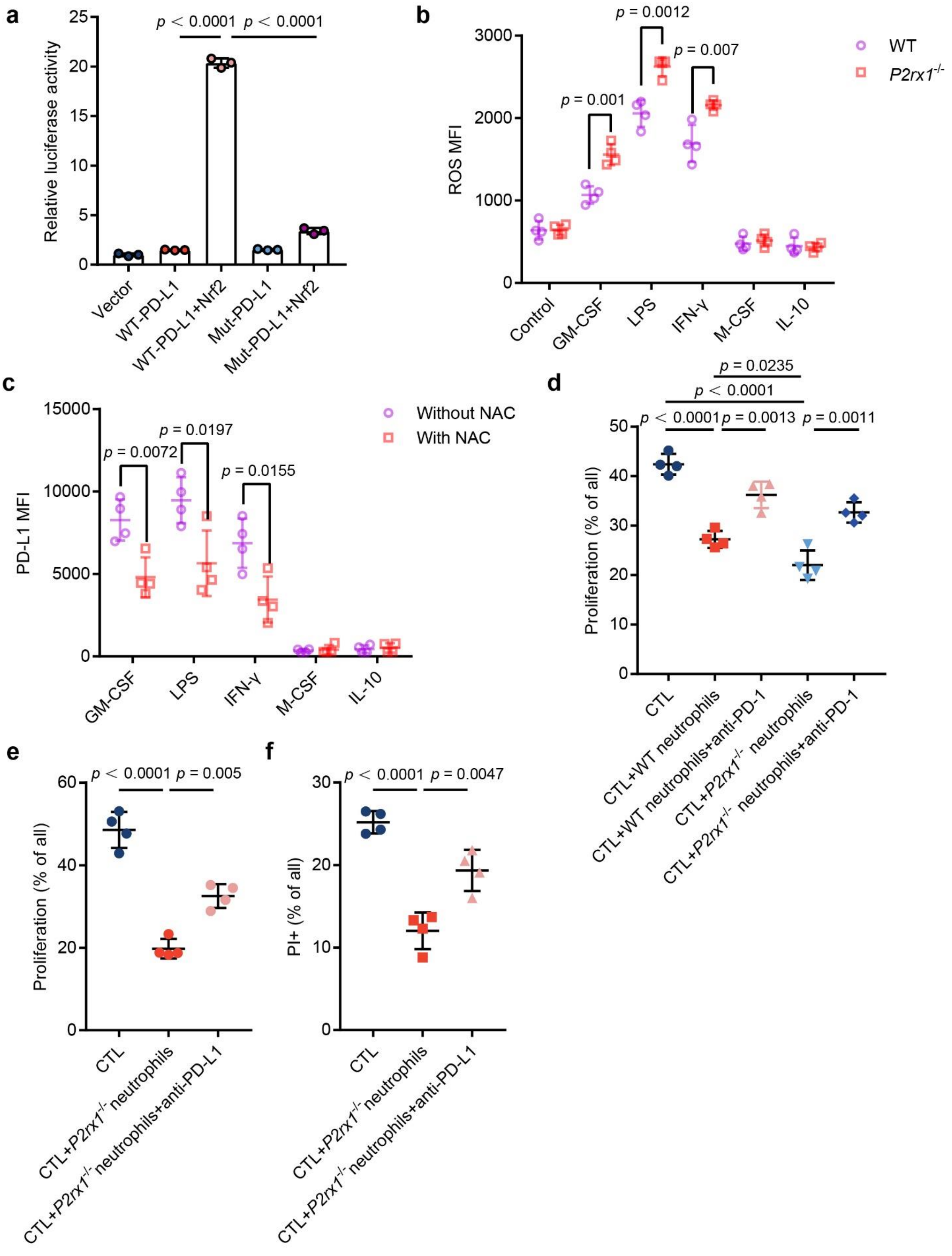
a
Relative luciferase activity
p < 0.0001
p < 0.0001
Vector
WT-PD-L1
WT-PD-L1+Nrf2
Mut-PD-L1
Mut-PD-L1+Nrf2
b
ROS MFI
p = 0.0012
p = 0.007
p = 0.001
WT
P2rx1-/-
Control
GM-CSF
LPS
IFN-γ
M-CSF
IL-10
c
PD-L1 MFI
p = 0.0072
p = 0.0197
p = 0.0155
Without NAC
With NAC
GM-CSF
LPS
IFN-γ
M-CSF
IL-10
d
Proliferation (% of all)
p = 0.0235
p < 0.0001
p < 0.0001
p = 0.0013
p = 0.0011
CTL
CTL+WT neutrophils
CTL+WT neutrophils+anti-PD-1
CTL+P2rx1-/- neutrophils
CTL+P2rx1-/- neutrophils+anti-PD-1
e
Proliferation (% of all)
p < 0.0001
p = 0.005
CTL
CTL+P2rx1-/- neutrophils
CTL+P2rx1-/- neutrophils+anti-PD-L1
f
PI+ (% of all)
p < 0.0001
p = 0.0047
CTL
CTL+P2rx1-/- neutrophils
CTL+P2rx1-/- neutrophils+anti-PD-L1

Supplementary Fig. 10. P2RX1- neutrophils inhibit CD8+ T cells activation. (a) WT or mutant PD-L1 was cloned into a luciferase reporter plasmid and co-transfected into NIH3T3 cells with Nrf2 overexpression. The reporter activity was determined by a Dual-Glo luciferase assay system (n=3 per group, 2 independent experiments). (b-c) Bone marrow neutrophils were isolated from WT or *P2rx1*$^{-/-}$ mice and stimulated with indicated stimulus. ROS and PD-L1 expression were determined by flow cytometry (n=4 per group, 3 independent experiments). (d) Antigen activated CTLs was co-cultured with GM-CSF primed WT or *P2rx1*$^{-/-}$ neutrophils. Cell proliferation was analyzed with CSFE staining in the presence or absence of anti-PD-1 neutralizing antibody (n=4 per group, 3 independent experiments). (e-f) Antigen activated CTLs was co-cultured with GM-CSF primed WT or *P2rx1*$^{-/-}$ neutrophils. Cell proliferation (e) and cytotoxicity (f) was analyzed in the presence or absence of anti-PD-L1 neutralizing antibody (n=4 per group, 2 independent experiments). Bars represent mean ± standard deviation in a-f. *P* values are derived from two-sided Student's t test (b-c), or one-way ANOVA and Tukey's multiple comparisons test (a, d, e and f). Source data are provided as a Source Data file.

Supplementary Table 1. Characteristics of patients with PDAC liver metastasis.

Sample	Age	Gender
1	58	Male
2	65	Male
3	71	Male
4	82	Female
5	40	Male
6	50	Female
7	61	Male
8	68	Female
9	58	Male
10	58	Male
11	69	Female
12	75	Male
13	69	Male
14	69	Female
15	55	Male
16	53	Male
17	77	Male
18	60	Female
19	47	Male
20	58	Female

Supplementary Table 2. WT and mutant *PD-L1* plasmid**.**

WT *PD-L1* plasmid
AGAGACTCACCTGCCACTGGCTCCTGAGTACTGGAATTAAGGCGTGTGTCACCGCACCGAAGCCTAG<br>TTTCGTTTTTTCTTAAACTGTGAATATCCCAAAGCTGACTCTAAAGTCATCCGCAGGAAATACTATGAG<br>ATAAACTCATGCTCAAAGGGACTGGGTGGCTTCGGTTTCACAGACAGCGGAGGTTGGACAAGGCTTC<br>CGCGGAGTGGGCGGGGCTCTGAACTCGAGATAAGACCAGGAAATCGTGGTCCCCAAGCCTCATGCC<br>AGGCTGCACTTGCACGTCGCGGGCCAGTCTCCTCGCCTGCAGGTAAGGGAGCATCTTCTCGCGGAAT<br>CCGCTTGCAGGGCACTTTAAAGAGCCAGAATCCCTAGACCTTTTTAGGACGGAGAAGGGAACCGGTT<br>TCCTGGGAAAGTTAAGAACTCAGAATCCGCAGTTTTGTGTGTTTATGGATCTTGTGGGTAGGTAGCTG<br>GGTCAGAAGAGATGAATTAATTGGTCCTAGCGCGACTTGACTGTTTGCTAGCAATGACTGGGTCTTTC<br>CACTTGAAGCATCTCCGGAGGTCCCTTCCTCTGTCGAGGTCTAGGATGCTGGAGCTTAAGATTTTCAT<br>TCTATCTGCCCAGAGAACCTAAAGGATTTTTGGAAGAAAATGTCCCAAACAGTTCTTAGATACAGTGA<br>CCTAGGCTAT
Mutant *PD-L1* plasmid
AGAGACTCACCTGCCACTGGCTCCTGAGTACTGGAATTAAGGCGTGTGTCACCGCACCGAAGCCTAG<br>TTTCGTTTTTTCTTAAACTGTGAATATCCCAAAGCTGACTCTAAAGTCATCCGCAGGAAATACTATGAG<br>ATAAACTCATGCTCAAAGGGACTGGGTGGCTTCGGTTTCACAGACAGCGGAGGTTGGACAAGGCTTC<br>CGCGGAGTGGGCGGGGCTCTGAACTCGAGATAAGACCAGGAAATCGTGGTCCCCAAGCCTCATGCC<br>AGGCTGCACTTGCACGTCGCGGGCCAGTCTCCTCGCCTGCAGGTAAGGGAGCATCTTCTCGCGGAAT<br>CCGTGGATCTGGCACTTTAAAGAGCCAGAATCCCTAGACCTTTTTAGGACGGAGAAGGGAACCGGTT<br>TCCTGGGAAAGTTAAGAACTCAGAATCCGCAGTTTTGTGTGTTTATGGATCTTGTGGGTAGGTAGCTG<br>GGTCAGAAGAGATGAATTAATTGGTCCTAGCGCGACTTGACTGTTTGCTAGCAATGACTGGGTCTTTC<br>CACTTGAAGCATCTCCGGAGGTCCCTTCCTCTGTCGAGGTCTAGGATGCTGGAGCTTAAGATTTTCAT<br>TCTATCTGCCCAGAGAACCTAAAGGATTTTTGGAAGAAAATGTCCCAAACAGTTCTTAGATACAGTGA<br>CCTAGGCTAT
