# Supplementary material for: Identification of a subset of immunosuppressive P2RX1-negative neutrophils in pancreatic cancer liver metastasis
Source: Nat Commun. 2021 Jan 8;12:174. doi: 10.1038/s41467-020-20447-y (PMC7794439; doi:10.1038/s41467-020-20447-y)
Supplement: Supplementary file 3 — Reporting Summary [file 41467_2020_20447_MOESM3_ESM.pdf]

nature research

Corresponding author(s): Zhi-Gang Zhang, Xue-Li Zhang, Shu-Heng Jiang, Yong-Wei Sun

Last updated by author(s): Nov 19, 2020

Reporting Summary

Nature Research wishes to improve the reproducibility of the work that we publish. This form provides structure for consistency and transparency in reporting. For further information on Nature Research policies, see Authors & Referees and the Editorial Policy Checklist .

Statistics

For all statistical analyses, confirm that the following items are present in the figure legend, table legend, main text, or Methods section.

n/a	Confirmed	
☐	☒	The exact sample size (*n*) for each experimental group/condition, given as a discrete number and unit of measurement
☐	☒	A statement on whether measurements were taken from distinct samples or whether the same sample was measured repeatedly
☐	☒	The statistical test(s) used AND whether they are one- or two-sided<br>*Only common tests should be described solely by name; describe more complex techniques in the Methods section.*
☒	☐	A description of all covariates tested
☐	☒	A description of any assumptions or corrections, such as tests of normality and adjustment for multiple comparisons
☐	☒	A full description of the statistical parameters including central tendency (e.g. means) or other basic estimates (e.g. regression coefficient) AND variation (e.g. standard deviation) or associated estimates of uncertainty (e.g. confidence intervals)
☐	☒	For null hypothesis testing, the test statistic (e.g. *F*, *t*, *r*) with confidence intervals, effect sizes, degrees of freedom and *P* value noted<br>*Give P values as exact values whenever suitable.*
☒	☐	For Bayesian analysis, information on the choice of priors and Markov chain Monte Carlo settings
☒	☐	For hierarchical and complex designs, identification of the appropriate level for tests and full reporting of outcomes
☒	☐	Estimates of effect sizes (e.g. Cohen's *d*, Pearson's *r*), indicating how they were calculated

Our web collection on statistics for biologists contains articles on many of the points above.

Software and code

Policy information about availability of computer code

Data collection	Confocal microscopy images were taken using Leica SP8 LAS X. Pathological images were acquired using Leica Aperio ScanScope Console 12.3. Flow cytometry signals were detected in BD Fortessa FACS with FACSDiva software v6.0. Luciferin emission imaging was performed with Perkin Elmer LifeImage 4.5.5. Seahorse metabolic data were acquired with Agilent Wave 2.6.1. RNA sequencing data were collected using NextSeq System Suite v2.2.0.
Data analysis	Flow cytometry data was analyzed using FlowJo Software version 10.4.2 software. Seahorse metabolic data were analyzed with Agilent Wave 2.6.1. RNA sequencing data were analyzed with R-3.3.1. Single cell sequencing data was analyzed with Bioturing BBrowser 2.3.44. For statistical analysis and graphical data presentation we used GraphPad Prism 7.04.

For manuscripts utilizing custom algorithms or software that are central to the research but not yet described in published literature, software must be made available to editors/reviewers. We strongly encourage code deposition in a community repository (e.g. GitHub). See the Nature Research guidelines for submitting code & software for further information.

Data

Policy information about availability of data

All manuscripts must include a data availability statement. This statement should provide the following information, where applicable:

- Accession codes, unique identifiers, or web links for publicly available datasets
- A list of figures that have associated raw data
- A description of any restrictions on data availability

The PDAC liver metastases transcriptome data used in this study are available in the GEO database under accession code GSE71729 [https://www.ncbi.nlm.nih.gov/geo/query/acc.cgi?acc=GSE71729] and GSE151580 [https://www.ncbi.nlm.nih.gov/geo/query/acc.cgi?acc=GSE151580]. The RNA-seq data generated in this study have been deposited in the Sequence Read Archive (SRA) repository under accession code PRJNA664673 [https://www.ncbi.nlm.nih.gov/bioproject/PRJNA664673/]. Source data are available as a Source Data file. The remaining data are available within the Article, Supplementary Information or available from the authors upon request.

Field-specific reporting

Please select the one below that is the best fit for your research. If you are not sure, read the appropriate sections before making your selection.

- [x] Life sciences
- [ ] Behavioural & social sciences
- [ ] Ecological, evolutionary & environmental sciences

For a reference copy of the document with all sections, see nature.com/documents/nr-reporting-summary-flat.pdf

Life sciences study design

All studies must disclose on these points even when the disclosure is negative.

Sample size	Sample size was not predetermined with statistical methods, but based on expected effect size and variability within the sample, as well as cost and feasibility of experiments.
Data exclusions	No data were excluded from analysis.
Replication	Experiments involving in vitro cell studies and most of animal studies were repeated three times to ensure reproducibility. All attempts at replication were successful.
Randomization	All mice and cells were randomly assigned to experimental groups.
Blinding	Investigators were blinded to group allocation for mouse experiments. Also, quantification of tumor parameters by histological analyses was performed in a blinded fashion. For in vitro experiments, investigators were not blinded in conducting experiments as they needed to know which groups to treat with which drugs. Where possible, researchers were blinded during data analysis.

Reporting for specific materials, systems and methods

We require information from authors about some types of materials, experimental systems and methods used in many studies. Here, indicate whether each material, system or method listed is relevant to your study. If you are not sure if a list item applies to your research, read the appropriate section before selecting a response.

Materials & experimental systems

n/a	Involved in the study	
[ ]	[x]	Antibodies
[ ]	[x]	Eukaryotic cell lines
[x]	[ ]	Palaeontology
[ ]	[x]	Animals and other organisms
[ ]	[x]	Human research participants
[x]	[ ]	Clinical data

Methods

n/a	Involved in the study	
[x]	[ ]	ChIP-seq
[ ]	[x]	Flow cytometry
[x]	[ ]	MRI-based neuroimaging

Antibodies

Antibodies used	Antibodies used (name; company; catalog number; clone; dilution): P2RX1 (Alomone, APR-022, 270-283,1:80 for flow cytometry, 1:200 for WB); PerCP/Cyanine5.5 anti-mouse CD45 (eBioscience, 45-0451-82, 30-F11, 1994158, 1:100); PE anti-mouse Ly6G (Biolegend, 127607, 1A8, B276318, 1:100); PE/Cy7 anti-mouse Ly6G (Biolegend, 127617, 1A8, 13288786, 1:100); FITC anti-mouse PD-1 (eBioscience, 11-9981-82, RMP1-30, 4347279, 1:100); APC anti-mouse PD-L1 (Biolegend, 124311, 10F.9G2, 124311, 1:100); PE anti-mouse PD-L2 (Biolegend, 107205, TY25, B215549, 1:100); PE anti-mouse CD80 (BD, 561955, 16-10A1, 8184643, 1:100); PE anti-mouse CD86 (BD, 561963, GL1, 7075951, 1:100); Alex Fluor 488 anti-mouse F4/80 (eBioscience, MF48020, BM8, 1660871, 1:100); APC anti-mouse CD11C (Biolegend, 117309, N418, B216129, 1:100); Brilliant Violet 510 anti-mouse CD8a, (Biolegend, 100751, 53-6.7, 1:100); PerCP-Cyanine5.5 anti-mouse CD19 (eBioscience, 45-0193-80, 1D3, 4300340, 1:100); anti-mouse Nrf2 (CST, 12721, D1Z9C, 1:1000 for flow cytometry, 1:200 for ChIP); Alexa Fluor 594 anti-rabbit secondary antibody (Abcam, ab150080, GR3192711-2, 1:500 for flow cytometry, 1:1000 for immunofluorescence); anti-CitH3 (Abcam, ab5103, citrulline R2 + R8 + R17, 1:1000); anti-PD-1 (BioXCell, BE0146, RMP1-14, 20 μg/mL for neutralizing); anti-PD-L1 (BioXCell, BE0101, 10F.9G2, 20 μg/mL for neutralizing); anti-GM-CSF (Bioligand, 505415, MP1-22E9, 10 μg/mL for neutralizing); anti-CD66b (Biologend, 305102, G10F5. 1:1000 for immunofluorescence); anti-Ly6G (Biolegend, 127650, 1A8, 1:1000 for immunofluorescence, 12.5 μg/mice for neutrophil depletion).
Validation	Antibody validation information can be found on manufacturers' website:<br>anti-P2RX1: https://www.alomone.com/p/anti-p2x1-receptor-extracellular/APR-022<br>PerCP/Cyanine5.5 anti-mouse CD45: https://www.thermofisher.com/cn/zh/antibody/product/CD45-Antibody-clone-30-F11-Monoclonal/45-0451-82<br>PE anti-mouse Ly6G: https://www.biolegend.com/en-us/products/pe-anti-mouse-ly-6g-antibody-4777?GroupID=GROUP20<br>PE/Cy7 anti-mouse Ly6G: https://www.biolegend.com/en-us/products/pe-cyanine7-anti-mouse-ly-6g-antibody-6139?

GroupID=GROUP20
FITC anti-mouse PD-1: https://www.thermofisher.com/order/genome-database/generatePdf?productName=CD279%20(PD-1)&assayType=PRANT&detailed=true&productId=11-9981-82
APC anti-mouse PD-L1: https://www.biolegend.com/de-de/search-results/apc-anti-mouse-cd274-b7-h1--pd-l1-antibody-6655
PE anti-mouse PD-L2: https://www.biolegend.com/en-us/products/pe-anti-mouse-cd273-b7-dc-pd-l2-antibody-2547
PE anti-mouse CD80: https://www.bdbiosciences.com/us/p/561955
PE anti-mouse CD86: https://www.bdbiosciences.com/us/p/561963
Alex Fluor 488 anti-mouse F4/80: https://www.thermofisher.com/antibody/primary/target/f4/80/application/Flow%20Cytometry
APC anti-mouse CD11C: https://www.biolegend.com/en-us/products/apc-anti-mouse-cd11c-antibody-1813
Brilliant Violet 510 anti-mouse CD8a: https://www.biolegend.com/en-gb/products/brilliant-violet-510-anti-mouse-cd8a-antibody-7992
PerCP-Cyanine5.5 anti-mouse CD19: https://www.thermofisher.com/antibody/product/CD19-Antibody-clone-eBio1D3-1D3-Monoclonal/45-0193-82
anti-Nrf2: https://www.cellsignal.com/products/primary-antibodies/nrf2-d1z9c-xp-rabbit-mab/12721
Alexa Fluor 594 anti-rabbit secondary antibody: https://www.abcam.com/goat-rabbit-igg-hl-alexa-fluor-594-ab150080.html
anti-CitH3: https://www.abcam.com/histone-h3-citrulline-r2--r8--r17-antibody-ab5103.html
anti-PD-1: https://bxcell.com/product/invivomab-anti-m-pd-1/
anti-PD-L1: https://bxcell.com/product/m-pdl-1/
anti-GM-CSF: https://www.biolegend.com/en-us/products/ultra-leaf-purified-anti-mouse-gm-csf-antibody-17650
anti-CD66b: https://www.biolegend.com/en-us/products/purified-anti-human-cd66b-antibody-667
anti-Ly6G: https://www.biolegend.com/en-us/products/ultra-leaf-purified-anti-mouse-ly-6g-antibody-7740

Eukaryotic cell lines

Policy information about cell lines

Cell line source(s)	HPNE, ASPC-1, BxPC-3, Capan-2, CFPAC-1 and HPAC were purchased from ATCC; FC1199 cell line was gifting from Professor Jing Xue (State Key Laboratory of Oncogenes andRelated Genes, Shanghai Cancer Institute, Ren Ji Hospital, School of Medicine, Shanghai Jiao Tong University).
Authentication	The purchased cell lines were validated by the commercial vendors, and FC1199 cell line was validated by Professor Jing Xue using short tandem repeat (STR) profiling.
Mycoplasma contamination	All cell lines were tested negative for mycoplasma contamination.
Commonly misidentified lines (See ICLAC register)	No misidentified cell line was used in the study.

Animals and other organisms

Policy information about studies involving animals; ARRIVE guidelines recommended for reporting animal research

Laboratory animals	KrasG12D/+;Trp53R172H/+;Pdx-1-Cre (KPC) mice were purchased from The Jackson Laboratory. P2RX1 knockout (P2rx1-/-) mice were purchased from GemPharmatech Co., Ltd, China. 20-26 weeks male or female KPC mice, and 6-8 weeks WT or P2rx1-/- mice male mice were used. Animals were housed in East China Normal University SPF animal facility, in temperatures 20-22ÿ, humidity 30-70% and a 12-hour light/12-hour dark cycle.
Wild animals	No wild animals were used in this study.
Field-collected samples	No field-collected samples were used in the study.
Ethics oversight	Animal experiments were approved by Institutional Animal Care and Use Committee of East China Normal University.

Note that full information on the approval of the study protocol must also be provided in the manuscript.

Human research participants

Policy information about studies involving human research participants

Population characteristics	This information is shown in supplementary table 1.
Recruitment	20 cases of PDAC liver metastases and adjacent livers tissues were obtained from Ren Ji hospital from January 2015 to June 2018. All patients had not received radiotherapy, chemotherapy, hormone therapy or other related anti-tumor therapies before surgery.
Ethics oversight	The study was approved by the Research Ethics Committee of Ren Ji Hospital, School of Medicine, Shanghai Jiao Tong University. Written informed consent was provided before enrollment.

Note that full information on the approval of the study protocol must also be provided in the manuscript.

Flow Cytometry

Plots

Confirm that:

- [x] The axis labels state the marker and fluorochrome used (e.g. CD4-FITC).
- [x] The axis scales are clearly visible. Include numbers along axes only for bottom left plot of group (a 'group' is an analysis of identical markers).
- [x] All plots are contour plots with outliers or pseudocolor plots.
- [x] A numerical value for number of cells or percentage (with statistics) is provided.

Methodology

Sample preparation	Mice were euthanized and liver metastases were harvested. Tissues were carefully minced and digested with 2 mg/mL collagenase A and 1×DNase I for 30 min. Digestion was quenched by FBS, and filtered with 40 μm Nylon mesh. Nonparenchymal cell were enriched by undergoing a centrifugation step through 37.5% Percoll. The cell pellets at the tube bottom were washed and resuspended in staining buffer (2%FBS in PBS). Single cell suspensions were blocked with anti-mouse CD16/CD32 antibodies for 10 min and labeled with indicated fluorophore-conjugated antibodies at recommended dilutions and times.
Instrument	BD Fortessa
Software	FACS Diva software and FlowJo were used to collect and analyze data.
Cell population abundance	Bone marrow neutrophils were obtained by centrifugation with discontinuous Percoll gradient (78 %, 69 % and 52 %) and harvested between the 78 % and 69 % layers, with cell purity greater than 93%. Metastatic tissue infiltrated neutrophils were isolated by FACS, with cell purity greater than 95%.
Gating strategy	Gating strategies are clearly described in the figure legends. Basically, live cells were gated with FCS and SSC and subsequently gated with various markers as indicated in the figures.

- [x] Tick this box to confirm that a figure exemplifying the gating strategy is provided in the Supplementary Information.
